# Hereditary spastic paraplegia SPG13 mutation increases structural stability and ATPase activity of human mitochondrial chaperonin

**DOI:** 10.1038/s41598-022-21993-9

**Published:** 2022-10-31

**Authors:** Lingling Chen, Aiza Syed, Adhitya Balaji

**Affiliations:** Department of Molecular and Cellular Biochemistry, Indiana University Bloomington, 212 S. Hawthorne Dr., Bloomington, IN 47405 USA

**Keywords:** Chaperones, Motor neuron disease

## Abstract

Human mitochondrial chaperonin mHsp60 is broadly associated with various human health conditions and the V72I mutation in mHsp60 causes a form of hereditary spastic paraplegia, a neurodegenerative disease. The main function of mHsp60 is to assist folding of mitochondrial proteins in an ATP-dependent manner. In this study, we unexpectedly found that mutant mHsp60^V72I^ was more stable structurally and more active in the ATPase activity than the wildtype. Analysis of our recently solved cryo-EM structure of mHsp60 revealed allosteric roles of V72I in structural stability and ATPase activity, which were supported by studies including those using the V72A mutation. Despite with the increases in structural stability and ATPase activity, mHsp60^V72I^ was less efficient in folding malate dehydrogenase, a putative mHsp60 substrate protein in mitochondria and also commonly used in chaperonin studies. In addition, although mHsp60^V72I^ along with its cochaperonin mHsp10 was able to substitute the *E. coli* chaperonin system in supporting cell growth under normal temperature of 37 °C, it was unable under heat shock temperature of 42 °C. Our results support the importance of structural dynamics and an optimal ATP turnover that mHsp60 has evolved for its function and physiology. We propose that unproductive energy utilization, or hyperactive ATPase activity and compromised folding function, not mutually exclusive, are responsible for the V72I pathology in neurodegenerative disease.

## Introduction

Mitochondria are essential for eukaryotic cells. One of the essential functions is to generate energy in the form of ATP: as the cellular power house, mitochondria provide more than 90% of the energy the human body needs to function^1^. Another essential function is to coordinate metabolic processes. Mitochondria produce metabolites for biosynthesis of amino acids, nucleotides, and fatty acids that are essential for both cell growth and communication^2,3^. A third essential function is to maintain Ca^2+^ homeostasis^4^: Ca^2+^ as an universal second messenger regulates a range of cellular activities including the synaptic activity in neurons^5^. These three essential mitochondrial functions are particularly important to neurons, as such, impaired mitochondrial function has been found associated with neurodegenerative disorders^6^.

Normal mitochondrial function relies on mitochondrial protein homeostasis, which is maintained by a range of molecular chaperones, for example, including mHsp60. mHsp60 belongs to a group of proteins called chaperonins that are highly conserved among the three kingdoms of life^7^ and are required for cellular viability under both normal and stress conditions^8^. The main function of chaperonins is to assist folding and assembly of cellular proteins using an ATP mechanism^9–13^. Broadly, mHsp60 has been associated with human health conditions including Alzheimer’s disease^14^, diabetes^15^, cancers^16^ and cardiovascular conditions^17^. Mutations in mHsp60 are responsible for neurodegenerative diseases^18^ including hereditary spastic paraplegia^19^ and MitCHAP60 disease^20^. The hereditary spastic paraplegias (HSP) are a large group of inherited neurologic disorders with the primary symptom of difficulty in walking due to muscle weakness and muscle tightness (spasticity) in the legs. More than 80 different genetic loci with over 60 gene products identified, naming *spastic paraplegia gene* (SPG) 1–83^21,22^.

**Figure 1:**
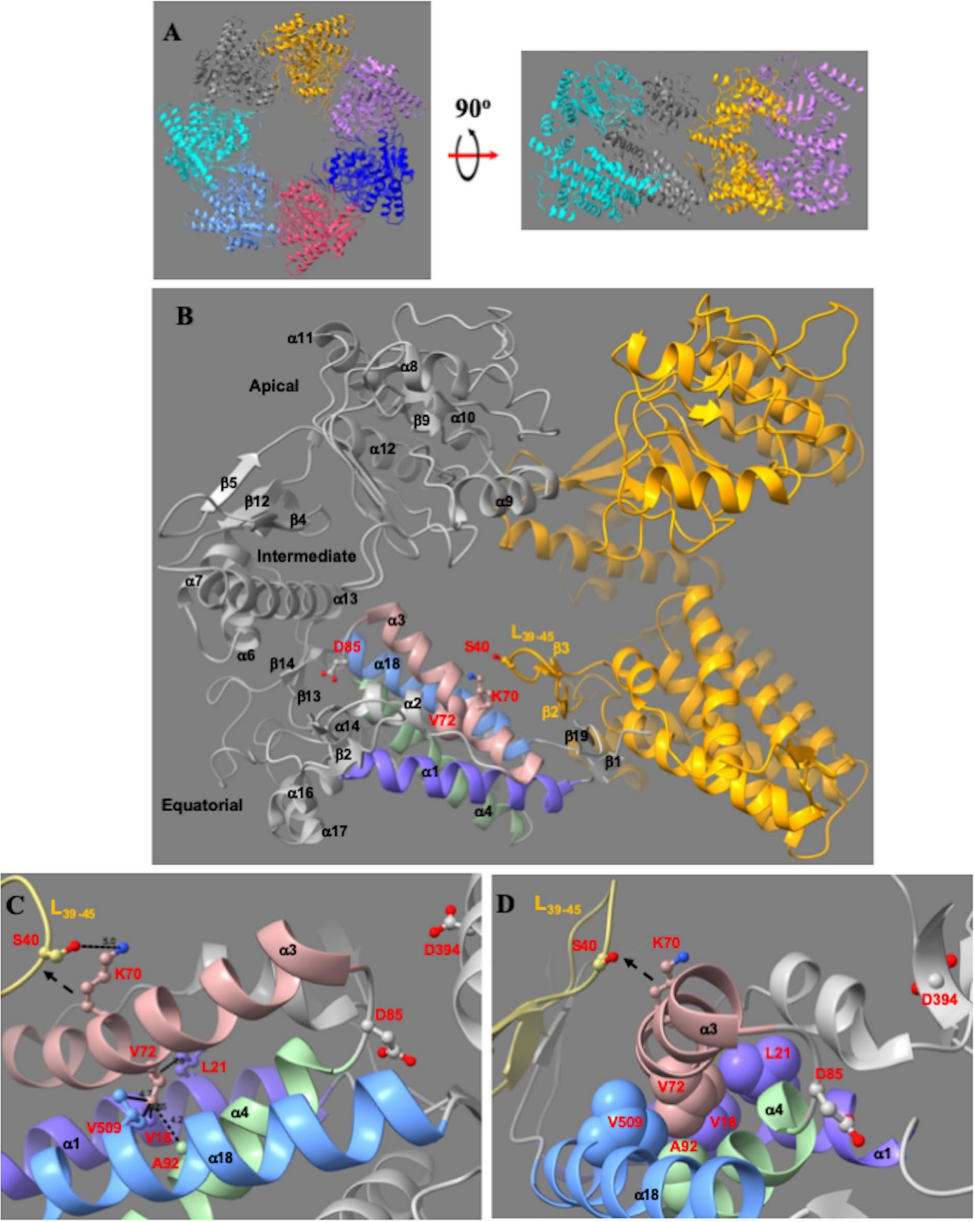
The proposed allosteric role of V72I mutation on the structural stability and the ATPase activity. (**A**) Overall structure of the mHsp60 heptamer. Left, view from the apical domains down to the equatorial domains; right, a sectional view of the sideview of the heptamer after removing three front subunits. Subunits in gray and gold are annotated in B). (**B**)The loop prior to α3 where V72 is located contains D85, the residue important for the ATP activity. The C-terminus of α3 is close to the inter-subunit interface: K70 on α3 of one subunit (gray) is near S40 from the neighboring subunit (gold). (**C**)V72 serves as the critical point where α3 (pink) transverses the α1-α4-α19, purple, green and blue, respectively, helical bundle. The V72-interacting residues are shown with sidechains, and distances between the sidechain of V72 and those of the interacting residues are shown and summarized in Table 3. Like D85, D394 is also important for the ATP activity. (**D**) Sidechain of V72 is embedded in a hydrophobic pocket in the three-helical junction. The ball-and-stick representation shows that sidechain of V72 is in direct van-der Waals contact with those of V18 and L21 (α1), A95 (α4) and V509 (α18). V72I mutation, mutation to a large residue, most likely shifts helix α3 towards the S40-containing loop of the neighboring subunit, diagramed with dashed arrows, enhancing the inter-subunit interaction and the structural stability.

SPG13 is caused by the V72I mutation in *mhsp60* gene^19^. mHsp60 is homo-heptameric with the seven subunits forming a ring-like structure (Fig. 1A)^23^. Each subunit consists of three domains, the apical, intermediate and equatorial domains (Fig. 1B). The equatorial domain contains the ATP binding site, and forms most of the inter-subunit interaction. Our recent structural analysis shows that the inter-subunit interactions in mHsp60 are weaker than those in the bacterial homolog GroEL^23^, providing structural basis for the intrinsic dissociation of the mHsp60 heptamer^24,25^. Notably, the SPG13 HSP associated residue V72 is located within the equatorial domain: it is in the middle of a long helix, the 22-a.a. α3 (Fig. 1B–C). In this study, we found that the V72I mutation impacted the protein’s structure and activities in unexpected manners. We offered structural explanations for the unexpected mutational effects, which were supported by our further mutational studies. Our studies allow us to propose two mechanisms, not mutually exclusive, for the V72I pathology.

## Results

### V72I mutation increased structural stability

**Figure 2:**
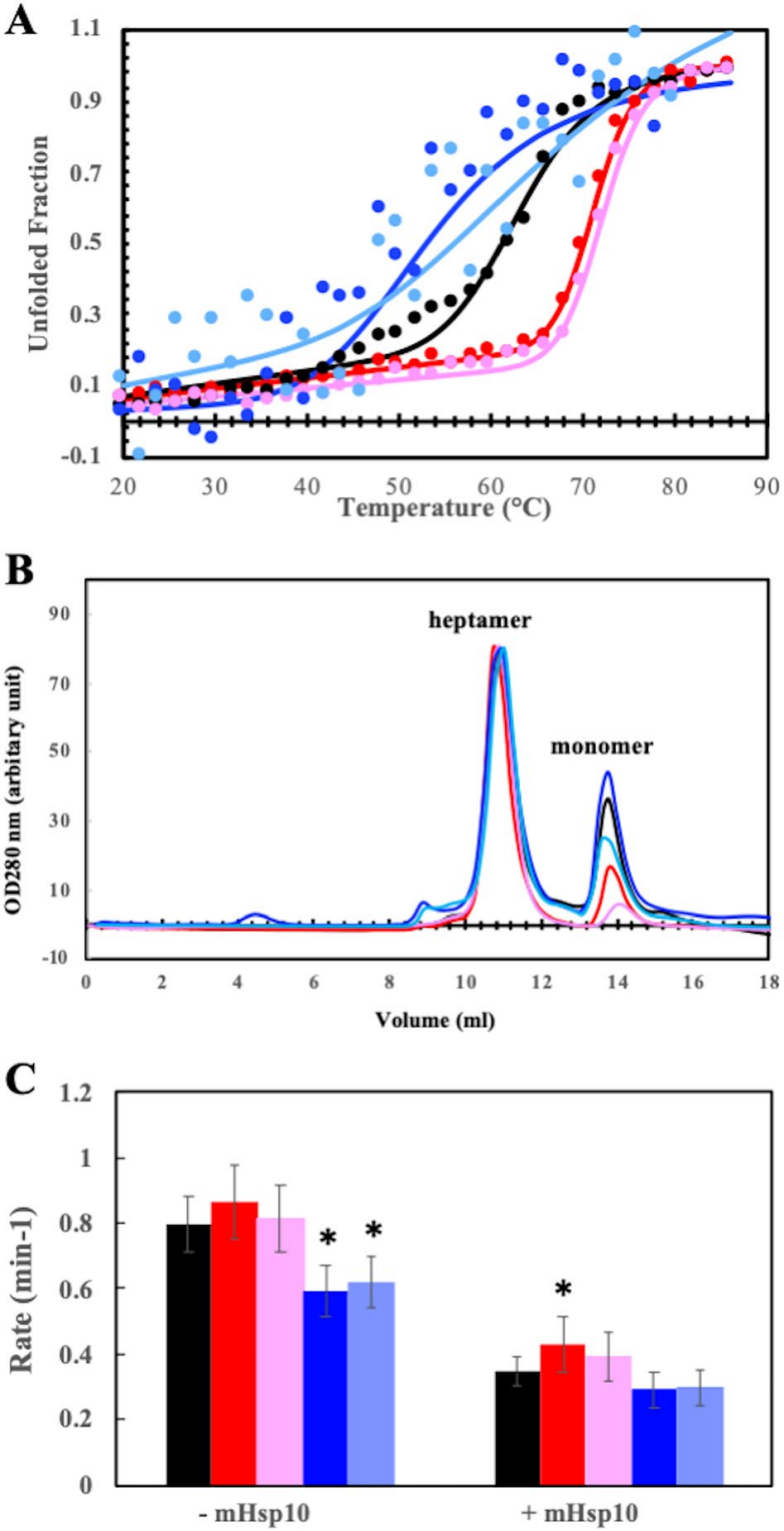
V72I mutation increased structural stability and ATPase activity. (**A**) Representative thermal unfolding of mHsp60 proteins. Unfolded fraction is defined as (y-y_N_)/(y_U_-y_N_) where y, y_N_, and y_U_ are the molar ellipticity (ϑ_222_) derived in Eq. (1). The solid lines are non-linear regression analysis using Eq. (1). The derived ∆H_vh_ and T_m_ values are listed in Table 1. (**B**)Size exclusion chromatographic profiles of mHsp60 proteins 96 h after purification at 4 °C. The profiles were scaled to the heptameric peak (V = 10.8 ml). (**C**)The ATPase activity of mHsp60 proteins in the absence and presence of cochaperonin mHsp10. Rates of ATPase activity, errors of standard deviation, the number of measurement, and p-value associated with one-way analysis of variance are listed in Table 2. ^*^p-value < 0.05. Wild type mHsp60 (black), mHsp60^V72I^ (red), mHsp60^V72I/GMless^ (pink), mHsp60^V72A^ (blue) and mHsp60^V72A/GMless^ (light blue).

The heptameric mHsp60 is highly dynamics, and readily dissociates into lower oligomeric states mainly monomers^24,25^. We first examined whether the pathological mutation affect mHsp60’s structural stability by studying the mutant’s thermal unfolding behaviors via circular dichoism (CD). Unexpectedly, mHsp60^V72I^ was more stable than the wildtype mHsp60 because the mutant had a higher melting temperature (T_m_) and a larger unfolding enthalpy (∆H_unfolding_) than mHsp60 (Fig. 2A, Table 1). To confirm the increased stability effect, we removed the last 26 a.a. fragment mainly consisting of the Gly-Met repeats, which is not visible in the CryoEM structure of mHsp60^23^, to generate a C-terminal truncation mHsp60^V72I/GMless^. Consistently, mHsp60^V72I/GMless^ had a higher T_m_ and larger ∆H_unfolding_ than mHsp60 (Fig. 2A, Table 1). In addition, the inter-subunit interactions in heptameric mHsp60^V72I^ and mHsp60^V72I/GMless^ were more stable than the wildtype because their time course dissociations were reduced when compared with the wildtype (Fig. 2B). Thus, the increased structural stability of the V72I mutants as seen via thermal unfolding is likely due to the increase in their subunit associations.

**Table 1:** Thermodynamic parameters from thermal unfolding of mHsp60 proteins via circular dichoism (CD).

Proteins	∆H_vh_ (kcal/mol)^1^	T_m_^2^ (^o^C)
mHsp60	7.81	63.6
mHsp60^V72I^	18.92	71.2
mHsp60^V72I/GMless^	18.47	72.2

^1^van’t Hoff enthalpy, derived from fitting data to Eq. (1); ^2^the temperature of the transition midpoint, derived from fitting data to Eq. (1).

### V72I mutation increased ATPase activity

Chaperonins use ATP to assist protein folding; we next examined whether the V72I mutation affected mHsp60’s ATPase activity. As shown in Fig. 2C, Table 2, F72I mutation appeared to increase the ATPase activity of mHsp60 modestly. In the presence of their own cochaperonins, chaperonins display decreased ATPase activities. For example, mHsp60 displays 50% decrease in the ATPase activity in the presence of mHsp10 (^23,26–28^, also Fig. 2C, Table 2). Similarly, in the presence of mHsp10, the ATPase activity of mHsp60^V72I^ decreased ~ 50%. Importantly, in the presence of mHsp10, mHsp60^V72I^ had significantly higher ATPase activitythan wildtype mHsp60, based on the one-way analysis of variance.

**Table 2:** ATPase activities of mHsp60 proteins in the absence and presence of mHsp10.

	− mHsp10 (min^−1^)^a^	+ mHsp10 (min^−1^)^a^
mHsp60	0.79 ± 0.07 (14)	0.34 ± 0.05 (10)
mHsp60^V72I^	0.86 ± 0.10 (10; 0.0927)^b^	0.45 ± 0.06 (6; 0.0060)
mHsp60^V72I/GMless^	0.81 ± 0.10 (8; 0.9483)	0.39 ± 0.08 (5; 0.5359)
mHsp60^V72A^	0.60 ± 0.08 (6; 0.0002)	0.29 ± 0.05 (4; 0.5487)
mHsp60^V72A/GMless^	0.62 ± 0.08 (7; 0.0009)	0.30 ± 0.05 (4; 0.6755)

^a^rates are expressed for per mHsp60 subunit, ^b^errors are standard deviations calculated from multiple measurements; number of the measurement and p-value from the one-way analysis of variance for the V72 mutant vs. mHsp60 are indicated within the parentheses.

Interestingly, the 32% increase in ATPase activity of mHsp60^V72I^ in the presence of mHsp10 contrasts with an early study, which reported that the ATPase activity of mHsp60^V72I^ is reduced to 31% of the wildtype in the presence of mHsp10^29^. In the study, mHsp60s including wildtype and mHsp60^V72I^ were purified via refolding and reconstitution steps, and the ATPase activities of mHsp60s in the absence of mHsp10 were not reported. A different study shows that wildtype mHsp60 did not have ATPase activity in the presence of mHsp10 without the unfolded substrate^30^. These studies highlight the difficulties in mHsp60 purification and the importance of sample validation. Thus, the difference in sample preparation most likely contributes to the discrepancy of the mHsp60^V72I^ ATPase activity between the early^29^ and our studies.

### Structural analysis of V72I mutation

The observed V72I mutation effects on increasing structural stability and ATPase activity are unique because mutations usually destabilize structure and decrease biochemical activities such as the ATPase activity. To understand the V72I mutational effect, we analyzed the mHsp60 structure we recently determined. V72 is situated at a junction where four long helices, α1, α3, α4 and α18, intercept via hydrophobic interactions. The four helical bundle extends across the mHsp60 equatorial domain diagonally, comprising more than 50% of the domain (Fig. 1B). Specifically, α3 transverses helices α1, α4 and α18, and the four helices intercept at V72. As shown in Fig. 1C–D and Table 3, V72 is lodged via direct van der Waals contacts with V18 (3.5 and 3.5 Å) and L21 of α1 (3.5 Å), A94 of α4 (4.2 Å), and V509 of α18 (4.7 Å). Such precise hydrophobic interactions lock the positions and orientations of the four helices particularly α3 because V72 is the residue anchoring α3 over the α1- α4- α18 three helical bundle. Importantly, the α3 N-terminus is in close proximity to a loop, L_39-45_, from the neighboring subunit: sidechain of K70 is 5.0 Å away from that of S44 from the neighboring subunit (Fig. 1B–D). In addition, the α3 C-terminus is associated with the nucleotide binding site, because the short 2-a.a. loop connecting α3 and α4 contains D85 (Fig. 1B–C), the key residue for ATP activity. The D85 equivalent in *E. coli* homolog GroEL, D87, coordinates directly with Mg^2+^ in the nucleotide binding site^31^, and is essential for ATP hydrolysis^32^.

**Table 3:** Atomic distances between V72 of α3 and the surrounding residues of three helices.

V72 sidechain atom	Interacting sidechain atom	Distance (Å)
V72Cγ1 (α3)	V18Cγ 2 (α1)	3.5
	A94Cβ (α4)	4.2
	V509Cγ1 (α18)	4.7
V72Cγ2 ( α )	V18Cγ2 (α1)	3.5
	L21Cδ2 (α1)	3.5

The above structural analysis shows that V72 plays a crucial structural role in anchoring and positioning α3 and that the ends of α3 are associated with the inter-subunit interactions and the ATPase activity. When V72 is mutated to a residue larger than Val such as Ile as in the V72I mutant, α3 most likely moves towards L_39-45_ of the neighboring subunit (Fig. 1C–D), promoting the inter-subunit interactions, for example, the charge-charge interactions between K70 and S44. Such structural analysis can explain the decreased heptameric dissociations (Fig. 2B) and the increased overall structural stabilities of mHsp60^V72I^ and mHsp60^V72I/GMless^ (Fig. 2A) compared to wildtype mHsp60. Structural basis for the increased ATPase activity, however, is not forthcoming, mainly because the overall ATPase activity is a result of several molecular steps including ATP binding, hydrolysis and release of ADP and Pi. To support that the size of residue at position 72 plays an important role in allostery of subunit association and ATPase activity, we mutated V72 to Ala, a residue smaller than Val, and we expected the V72A mutation to have effects opposite to V72I on both structural stability and ATPase activity.

As expected, the V72A mutation decreased the structural stability and ATPase activity. mHsp60^V72A^ dissociated to a larger extent than wildtype mHsp60 and mHsp60^V72I^ after an extended time (Fig. 2B), consistent with the decreased inter-subunit interactions as α3 shifts closer to the α1- α4- α18 helical bundle, resulting in its shifting away from the L_39-45_ of the neighboring subunit (Fig. 1C–D). We noticed that the removal of the GM-tail appeared to stabilize the structure, as shown in comparing mHsp60^V72I^ with mHsp60^V72I/GMless^ and mHsp60^V72A^ with mHsp60^V72A/GMless^; however, we were not able to offer explanations. Unlike the cooperative thermal unfolding of the wildtype and the V72I mutants, unfolding of the V72A mutants was gradual and noncooperative (Fig. 2A), indicating weak intramolecular interactions associated with the overall structural stabilities of the V72A mutants. Figure 2C shows that the ATPase activities of mHsp60^V72A^ alone and in the presence of mHsp10 appeared somewhat lower than those of wildtype mHsp60 and mHsp60^V72I^. Together, these V72 mutational results support our above allosteric model of V72 on the structural stability and ATPase activity.

### V72I mutation slowed the refolding kinetics of MDH

**Figure 3:**
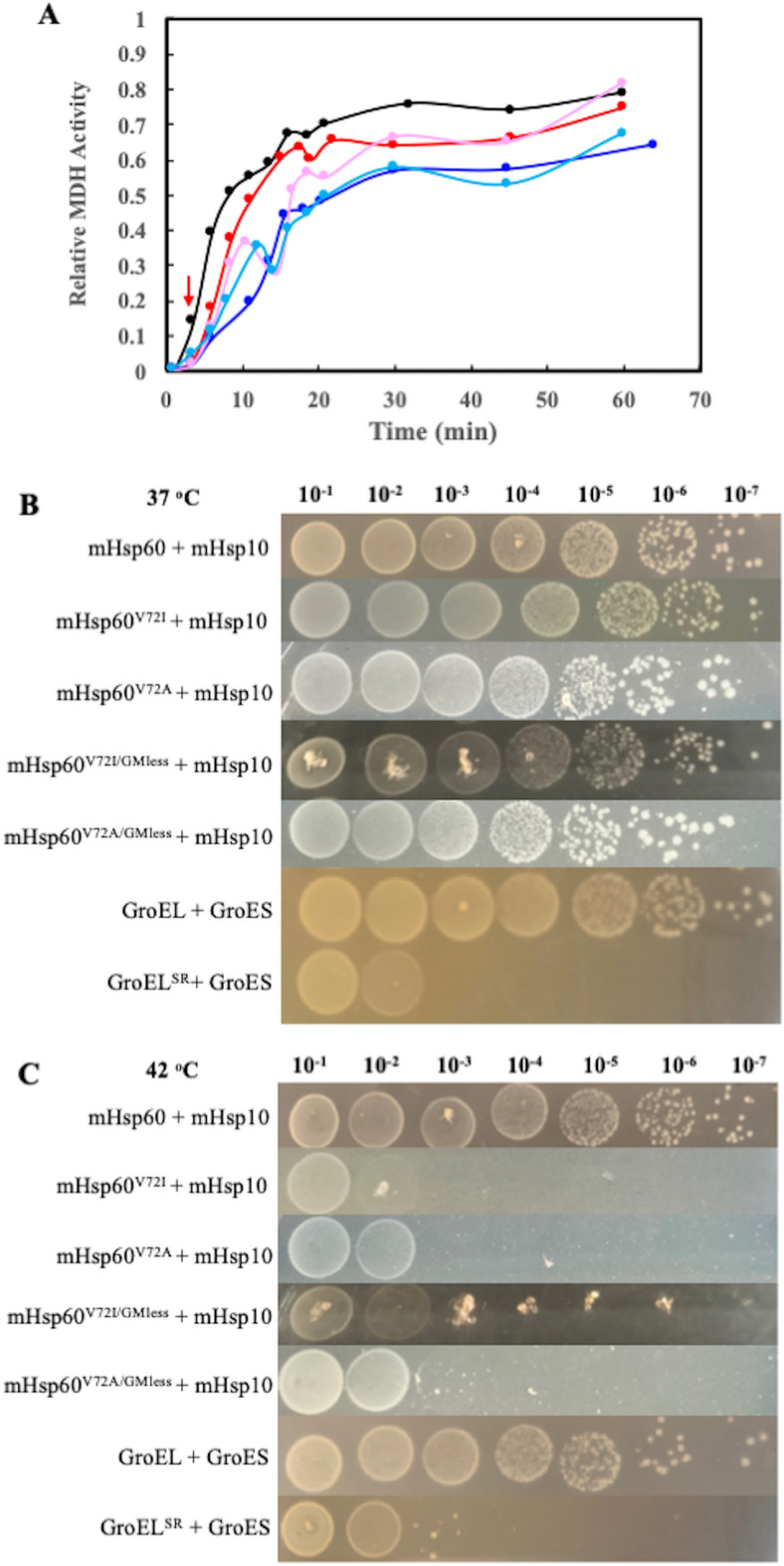
V72I mutation compromised both in vitro and in vivo folding activity of mHsp60. (**A**) Representatives of in vitro folding of MDH by wildtype mHsp60 and V72 mutants with cochaperonin mHsp10. Wild type mHsp60 (black), mHsp60^V72I^ (red), mHsp60^V72I/GMless^ (pink), mHsp60^V72A^ (blue) and mHsp60^V72A/GMless^ (light blue). The red arrow points to t = 3.5 min. (**B-C**)In vivo chaperone function of mHsp60 and V72 mutants at normal 37 °C (**B**) and heat stress 42 °C (**C**) conditions via assaying growth of *E*. *coli* MGM100 cells. mHsp60-mHsp10 supported the cell growth as well as GroEL-GroES. GroEL^SR^, a nonviable single-ring GroEL with GroES^42,43^, was used as a negative control.

Since the V72I mutation increased both the structural stability and the ATPase activity, we expected that the mutation also improved its assistance in folding proteins. Malate dehydrogenase (MDH) is presumably the mHsp60 substrate because it is one of the proteins identified that interact with mHsp60^33^. It is also commonly used for assessing the folding activity of chaperonins because its spontaneous folding is minimal^34^. Unexpectedly, mHsp60^V72I^, along with mHsp10, did not refold MDH better than wildtype mHsp60 (Fig. 3A). At 3.5 min upon initiating MDH refolding, ~ 15% of MDH achieved the functional folded state with the presence of mHsp60 while only 3% of MDH was folded with the presence of mHsp60^V72I^. The delay of mHsp60^V72I^ in refolding MDH continued until 13.5 min. In addition, the refolding yield at 60 min with mHsp60^V72I^ was slightly lower than the wildtype. The slow kinetics of MDH folding was also observed in mHsp60^V72I/GMless^ (Fig. 3A), confirming the defective effects of the V72I mutation in assisting MDH folding. Figure 3A also shows that the V72A mutation decreased both the folding kinetics and folding yield of MDH to more than the V72I mutation. Thus, although the V72I mutation increased both the structural stability and ATPase activity of mHsp60, the mutation slowed the MDH refolding kinetics and decreased the MDH refolding yield.

### V72I mutation diminished cell growth under heat stress

To examine the mutation effect on the chaperone function of mHsp60, we evaluated the abilities of the mutants along with mHsp10 to substitute the *E. coli* chaperonin GroEL and cochaperonin GroES in supporting *E. coli* cell growth under the normal (37 °C) and heat stress (42 °C) conditions. mHsp60/mHsp10 could substitute GroEL/GroES to support cell growth under both normal and stress conditions. At 37 °C, both V72I and V72A mutants, in either the full length or truncated form, supported cell growth just like the wildtype mHsp60 (Fig. 3B). On the contradictory, none of the V72 mutants were able to support cell growth under heat stress condition (Fig. 3C). The mutants’ loss of functions under the stress condition are consistent with their slow kinetics and their low yield in refolding MDH.

## Discussion

In this study, we examined the effects of the pathological V72I mutation and we provided structural mechanisms for the mutational effects. We found two striking unconventional effects caused by the V72I mutation. First, unlike the conventional mutations that destabilize protein structure and decrease protein activities, the V72I mutation increased both the structural stability and the ATPase activity. Second, unlike the conventional notions that increased structural stability and/or ATP hydrolysis rate enhance protein function, the V72I mutation compromised the in vitro and in vivo chaperone function. In addition, our structural and mutational analysis uncovered the allosteric mechanisms for the unexpected effects of the V72I mutation in structural stability and ATPase activity. Our studies allow us to propose mechanisms for the V72 mutation on the pathology of neurodegenerative disease hereditary spastic paraplegias SPG13.

Our studies highlight the unique properties, structural dynamics and optimal ATP turnover efficiency, that human mitochondrial mHsp60 has evolved. Unlike the paradigm bacterial chaperonin GroEL, mHsp60 is intrinsically dynamic as evident by its irreversible dissociation to monomers^24,25^. In our recent structural analysis^23^, we found that mHsp60 contains unique sequences that weaken the inter-subunit interaction, accounting for dynamics of the subunit association. In this study, we found that the V72I mutation fortuitously fosters inter-subunit interactions including a potential charge-charge interaction between K74 and S44, stabilizing the subunit association. The more stable mHsp60^V72I^ provides a unique opportunity to test the importance of structure dynamics of subunit association in mHsp60’s function to assist protein folding. Unexpectedly, we showed that mHsp60^V72I^ was less efficient (slow kinetic) in assisting folding of MDH, a presumptive endogenous substrate of mHsp60 in mitochondria^33^. In addition, we showed that mHsp60^V72I^ lost the chaperone function in supporting cell growth under heat stress. These compromised in vitro and in vivo mHsp60 functions, together with the HSP SPG13 pathology caused by the V72I mutation^19^, underscore the critical role of subunit association dynamics in mechanism, function and physiology of mHsp60.

Subunit association dynamics has been proposed to account for the new locations and functions implicated for mHsp60, which may contribute to the V72I pathology. Besides its conventional location of mitochondrion, mHsp60 has been found on the outer surface of cells, in cellular compartments such as peroxisome and endoplasmic reticulum, and in biological fluids including blood^16,35–37^. The extra-mitochondrial locations may impart novel, location-specific functions to mHsp60. Although information on how mHsp60 reaches these new locations and what function-structure of mHsp60 is limited, monomeric mHsp60 has been proposed as a functional conformation for the new functions^36,38^. Thus, in addition to the importance in the main function in assisting protein folding, dynamics of mHsp60 subunit association may have mechanistic implications for its extra-mitochondrial functions, adding another complexity to the physiology of mHsp60.

Our studies show that mHsp60 has evolved to utilize ATP optimally in assisting protein folding. The ATPase activity of mHsp60-mHsp10 is ~ 25% of that of *E. coli* GroEL-GroES^23,26–28^; however, the efficiency and effectiveness of mHsp60-mHsp10 in assisting MDH folding are the same as GroEL-GroES^23,26^. In our previous studies to activate single-ring GroEL mutant GroEL^SR^ via mutations in a concatenated GroES GroES^7^, we found that the ATPase activities of the single-ring GroEL^SR^-GroES^7^ systems are not directly correlated with the folding activities of MDH^39^. For example, the ATPase activities of the systems with most active MDH folding activities are ~ 25% of the highest ATPase activities tested. Clearly, an optimal, energy efficient chaperonin system should assist protein folding at fast kinetics, with high yields, and at low ATP activity (ATP turnover rate). As such, mHsp60^V72I^ is biochemically an inefficient chaperonin in two folds. Like the conventional mutants, it is compromised in assisting protein folding; unlike the conventional mutants, it is unproductive in using more ATP molecules. Physiologically, the V72I mutation is pathological in two folds. Due to mHsp60’s essential role in maintaining protein homeostasis in mitochondria thereby mitochondrial function, the mutant’s compromised chaperone function impairs the overall mitochondrial function. In addition, its hyperactive ATP turnover drains the cellular ATP supplies including those in neuron cells. Neurons have high demand for ATP: they spend ~ 50% of the total consumed ATPs on the synaptic activity. Together, these two mechanisms, not mutually exclusive, may account for the V72I-associated HSP SPG13 neurodegenerative disease. To provide structural rational to intervene the pathological effects caused by the V72I mutation, we are investigating structural basis for how the V72I mutation increases ATPase activity as well as to confirm the V72I effect on structural stability as proposed our current structural analysis.

## Methods

### Protein purification

Gene encoding the mature mHsp60 sequence (without the mitochondrial targeting signal) was synthesized with codons optimized for protein expression in *E. coli*, and was inserted into pBbE5a with NdeI/BamHI sites. To remove the C-terminal 26 a.a. Gly-Met repeats, primers (5’) AAACATATGGCTAAAGATGTGAAGTTT and (3’) TTTCTTAAGTTATGGGTCCTTCTCT were used to generate mHsp60^GMless^. mHsp60 mutants, mHsp60^F72I^, mHsp60^V72A^ and their truncated counterparts were generated using QuickChange (Qiagen). *E. coli* BL21DE3 cells were used to express wildtype and mutant mHsp60 proteins. Conditions for cell growth and protein purification were published previously^23^.

### ATPase assay

The steady-state ATP hydrolysis rate was measured using the malachite green assay, as described in our previous work^40^. Briefly, mHsp60 proteins were added to TEA buffer (50 mM triethanolamine, pH 7.5, 50 mM KCl, and 20 mM MgCl_2_) to a final concentration of 0.25 μM heptameric mHsp60s. The final concentration of mHsp10 was 0.3 μM. The solution was incubated at 25 °C for 10 min. The hydrolysis was initiated by addition of 100 mM pH 7.0 ATP to a final concentration of 2 mM and followed every 3 min for 21 min using the malachite green assay. Measurements were repeated multiple times for each sample as indicated in Table 2.

### MDH folding assay

Refolded MDH was assayed by measuring its ability to convert NADH to NAD^+^, as described in our previous work^40^. Briefly, MDH was unfolded in TEA buffer including 3 M GdmHCl to a final concentration of 36.7 μM (monomeric MDH). To refold MDH, 2.75 μl of unfolded MDH was diluted at 1:100 (v/v) to a final volume of 275 μl of refolding solution (at 30 °C) containing 50 mM TEA, pH 7.4, 50 mM KCl, 1 mM ATP, and 10 μM heptameric mHsp60s with 51 μM mHsp10. At desired time intervals, 20 μl of reaction solution was removed and mixed with 1 ml of NADH assay solution (50 mM TrisCl, pH 7.4, 10 mM DTT, 0.2 mM NADH, 1 mM ketomalonate), and absorption at 340 nm was taken to monitor the decrease of NADH. As a positive control, activity of 36.7 μM native MDH (monomeric concentration) was measured at the same time intervals and was taken as 100% activity. Measurements were repeated more than three times for each sample.

### Circular dichoism (CD)

mHsp60 protein stocks were in 100 mM NaF, 10 mM KHEPES, pH 7.4, 1 mM EDTA, 1 mM DDT and 5% glycerol. Proteins were tenfold diluted to 100 mM NaF, 10 mM KHEPES, pH 7.4, 1 mM EDTA and 1 mM TCEP. Samples were heated from 20 to 86 °C with a heating rate of 2.0 °C/min by a Jasco Peltier thermo control device. The CD signal at 222 nm was recorded by a J-715 CD spectrometer every 2 °C with a 4-s averaging time. Measurements were repeated more than once for each sample. Thermal denaturation was analyzed by fitting the data to a two-state model according to the following equation^41^:

1 $$y=\frac{{y}_{N}+{m}_{N}T+\left({y}_{D}+{m}_{D}T\right)\mathrm{exp}\left[{\Delta H}_{vh}/R(1/{T}_{m}-1/T)\right]}{1+\mathrm{exp}\left[{\Delta H}_{vh}/R(1/{T}_{m}-1/T)\right]},$$

where y is the observed CD signal at 222 nm, *y*_N_ and *y*_D_ are the derived signals for the native and denatured samples, and *m*_N_ and *m*_D_ are the derived baseline slopes for the native and denatured samples, T is the temperature, ∆H_*vh*_ is the van’ Hoff enthalpy, R is the gas constant and T_m_ is the temperature of the transition midpoint. Experiments were repeated at least twice.

### In vivo* complementary cell growth assay*

The MGM100 *E*. *coli* cell strain (kanamycin resistant, Kan^R^), whose *groE* promoter is replaced by a pBAD promoter, was obtained from the *E*. *coli* Genetic Stock Center at Yale University. *groEL* and *groEL*^*SR*^ are in pTrc (ampicillin resistant Amp^R^) , a *lac* promoter-based vector, while all *mhsp60* wildtype or mutants are in the *lac*-based vector pBbE5a (Amp^R^). *mhsp10* and *groES* are in the *lac*-based vector pBbE5c (chloramphenicol, Chl^R^). CaCl_2_ competent MGM100 cells were co-transformed with both plasmids and plated onto LB agar containing 50 μg/mL kanamycin, 100 μg/ mL ampicillin, 50 μg/mL chloramphenicol, and 0.2% w/v arabinose. Cells were serially diluted and plated, with added glucose to repress the chromosomal *groE* and added IPTG to express plasmid-encoded genes with lac promoters. Conditions for cell growth and titration are described previously^40^. Experiments were repeated at least three times.

## Data Availability

The datasets generated and/or analyzed during the current study are available in the GenBank repository with the submission ID of 2596912.
